# Upper-Limb Motion Recognition Based on Hybrid Feature Selection: Algorithm Development and Validation

**DOI:** 10.2196/24402

**Published:** 2021-09-02

**Authors:** Qiaoqin Li, Yongguo Liu, Jiajing Zhu, Zhi Chen, Lang Liu, Shangming Yang, Guanyi Zhu, Bin Zhu, Juan Li, Rongjiang Jin, Jing Tao, Lidian Chen

**Affiliations:** 1 Knowledge and Data Engineering Laboratory of Chinese Medicine School of Information and Software Engineering University of Electronic Science and Technology of China Chengdu China; 2 College of Electrical and Information Engineering Hunan University Changsha China; 3 Chengdu Chronic Diseases Hospital Chengdu China; 4 College of Health Preservation and Rehabilitation Chengdu University of Traditional Chinese Medicine Chengdu China; 5 College of Rehabilitation Medicine Fujian University of Traditional Chinese Medicine Fuzhou China

**Keywords:** feature selection, inertial measurement unit, motion recognition, rehabilitation exercises, machine learning

## Abstract

**Background:**

For rehabilitation training systems, it is essential to automatically record and recognize exercises, especially when more than one type of exercise is performed without a predefined sequence. Most motion recognition methods are based on feature engineering and machine learning algorithms. Time-domain and frequency-domain features are extracted from original time series data collected by sensor nodes. For high-dimensional data, feature selection plays an important role in improving the performance of motion recognition. Existing feature selection methods can be categorized into filter and wrapper methods. Wrapper methods usually achieve better performance than filter methods; however, in most cases, they are computationally intensive, and the feature subset obtained is usually optimized only for the specific learning algorithm.

**Objective:**

This study aimed to provide a feature selection method for motion recognition of upper-limb exercises and improve the recognition performance.

**Methods:**

Motion data from 5 types of upper-limb exercises performed by 21 participants were collected by a customized inertial measurement unit (IMU) node. A total of 60 time-domain and frequency-domain features were extracted from the original sensor data. A hybrid feature selection method by combining filter and wrapper methods (FESCOM) was proposed to eliminate irrelevant features for motion recognition of upper-limb exercises. In the filter stage, candidate features were first selected from the original feature set according to the significance for motion recognition. In the wrapper stage, k-nearest neighbors (kNN), Naïve Bayes (NB), and random forest (RF) were evaluated as the wrapping components to further refine the features from the candidate feature set. The performance of the proposed FESCOM method was verified using experiments on motion recognition of upper-limb exercises and compared with the traditional wrapper method.

**Results:**

Using kNN, NB, and RF as the wrapping components, the classification error rates of the proposed FESCOM method were 1.7%, 8.9%, and 7.4%, respectively, and the feature selection time in each iteration was 13 seconds, 71 seconds, and 541 seconds, respectively.

**Conclusions:**

The experimental results demonstrated that, in the case of 5 motion types performed by 21 healthy participants, the proposed FESCOM method using kNN and NB as the wrapping components achieved better recognition performance than the traditional wrapper method. The FESCOM method dramatically reduces the search time in the feature selection process. The results also demonstrated that the optimal number of features depends on the classifier. This approach serves to improve feature selection and classification algorithm selection for upper-limb motion recognition based on wearable sensor data, which can be extended to motion recognition of more motion types and participants.

## Introduction

### Background

The combination of wearable devices and wireless network technologies enables modern health care service providers to ubiquitously monitor patients out of hospital who require long-term exercise [1-3]. Motion recognition plays an important role in maintaining the intensity and quality of autonomous training with no or reduced supervision [4]. O'Brien et al [5] investigated the performance of action recognition based on signals collected by accelerometer, gyroscope, and barometer sensors in a mobile phone in a home setting for stroke patients. Zhang et al [6] proposed a fuzzy kernel motion classiﬁer to address the overlapping motion class issue caused by irregular motion samples performed by patients with different functional impairments. Cui et al [7] developed an automatic gait analysis system for stroke patients based on multimodal fusion architecture. Cai et al [8] investigated the feasibility of a support vector machine (SVM) classifier for motion recognition of the upper-limb exercises via surface electromyogram (sEMG) signals [8]. Huang et al [9] proposed a knowledge-driven multimodal activity recognition framework that exploits external knowledge to fuse multimodal data.

### Feature Selection in Motion Recognition

Most motion recognition methods are based on feature extraction and machine learning algorithms [10]. Time-domain and frequency-domain features are extracted from original time series data [11,12]. Castiblanco et al [13] exploited myoelectric signals (EMG) to identify finger and hand motions through pattern recognition techniques. Several methods for feature extraction, ranking, and classification from EMG signals were implemented, and the performance of motion identification was compared. Shawen et al [14] developed 4 classifiers that use accelerometer and gyroscope data collected by mobile phone from able-bodied individuals to detect falls in individuals with a lower limb amputation. A set of 40 features was computed from the original sensor data, and classifiers were trained to detect falls. Lin et al [15] used 2 sensors on the arm and wrist to collect acceleration and angular velocity of 6 types of upper-limb exercises performed by 13 volunteers. Motor features were used to train a back-propagation neural network (BPNN) algorithm for motion recognition. Wu et al [16] developed a method to identify upper-limb motion for community rehabilitation. The feature vector space was established by variance, mean absolute value, the fourth-order autoregressive, zero crossings, and root mean square. Various feature sets were extracted for classification.

Feature selection is an essential step to eliminate redundant or irrelevant features for specific classification task so as to deal with high-dimensional data [17,18]. Its task is to find the most representative feature subset from the original feature set. Ramezani et al [19] analyzed physical activity sensor features and activities with regard to indoor localization. Random forest (RF) was used to build a predictive model based on the most significant features. The study demonstrated that a subset of features can better distinguish between at-risk patients that can gain independence versus patients that will be rehospitalized. Wang et al [20] proposed 2 feature selection methods to improve activity recognition. Experimental results showed that the proposed methods reduce the dimensionality of the original feature space and contribute to the enhancement of overall recognition accuracy. Fang et al [21] compared feature selection methods based on interclass distance for human activity recognition in smart home environments. The experimental results showed that activity recognition accuracy is related to the feature set selected and an unsuitable feature set increases computational complexity and degrades activity recognition accuracy. Zhou et al [22] proposed a feature selection method for human motion recognition based on open human motion data. The experimental results showed that their feature selection method yields better recognition accuracy than nonfeature selection models.

Feature selection methods can be categorized into filter and wrapper methods [23]. For filter methods, the selection of the feature subset is independent of the classification algorithm. The feature fitness is evaluated via the statistical characteristics of the dataset, and the features with top ranking fitness are selected [24,25]. Banos et al [26] proposed a feature selection method for physical activity recognition using a feature quality group ranking via statistical criteria based on discrimination and robustness. Satisfactory results were achieved in both laboratory and seminaturalistic activity living datasets for real problems using several classification models. Hong et al [27] proposed a motion gesture recognition system via accelerometer (MGRA) implemented on mobile devices. The best feature vector including 27 items was selected using the minimal-redundancy-maximal-relevance criterion taking both static and mobile scenarios into consideration. The experimental results confirmed that MGRA can accommodate a broad set of gesture variations within each class and achieve higher accuracy than previous methods [28]. As for wrapper methods, the feature subset is selected simultaneously with the estimation of its goodness in a specific classification task [29,30]. Camargo and Young [31] implemented motion classiﬁcation from sEMG signals for prosthetic control by exploiting Chow-Liu trees for selecting features and evaluating 6 different classiﬁcation algorithms as the wrapping component [32]. The results demonstrated that feature selection is critical for improving classification accuracy. Xue et al [33] presented a novel wrapper feature selection algorithm that utilizes a generic algorithm to wrap an extreme learning machine to search for the optimum feature subset. Experiments were conducted on benchmark datasets and compared with 4 filter methods and 2 hybrid wrapper methods. The results revealed that the presented wrapper method is useful for feature selection problems and outperforms other algorithms in comparison. Chen and Chen [34] introduced a wrapper method to eliminate irrelevant features during classifier construction by introducing the cosine distance into SVM. The feature selection method has been applied to fault diagnosis of rolling element bearings and diagnosis of mild cognitive impairment. The results showed that the proposed method has great capacity for feature selection and pattern recognition.

### The Hybrid Feature Selection Method

Filter methods are often time-efficient, but the results are not always satisfactory. On the other hand, wrapper methods usually achieve better performance, but could be computationally intensive and the obtained feature subset optimized only for the specific learning algorithm [35]. As a result, hybrid feature selection methods take advantages of both filter and wrapper methods. Manbari et al [36] presented a hybrid feature selection algorithm based on the combination of clustering and the modified binary ant system to overcome the search space and high-dimensional data processing challenges. A damped mutation strategy was introduced to avoid local optima, and a new redundancy reduction policy was adopted to estimate the correlation between the selected features so as to further improve the algorithm.

Existing feature selection methods usually select feature sets that are relevant for specific classification tasks. To select the most representative features for motion recognition of upper-limb exercises, we propose a hybrid feature selection method combining the filter and wrapper methods called FESCOM in this paper. In the filter stage, candidate features are selected by ranking the feature significance index, which reflects the importance of each feature for motion recognition. In the wrapper stage, a classifier-specific feature selection algorithm is applied to further refine the candidate features. Classifiers including kNN, NB, and RF are constructed as the wrapping components. To the best of our knowledge, FESCOM is the first method that exploits hybrid feature selection for motion recognition of upper-limb exercises.

## Methods

### Workflow

**Figure 1:**
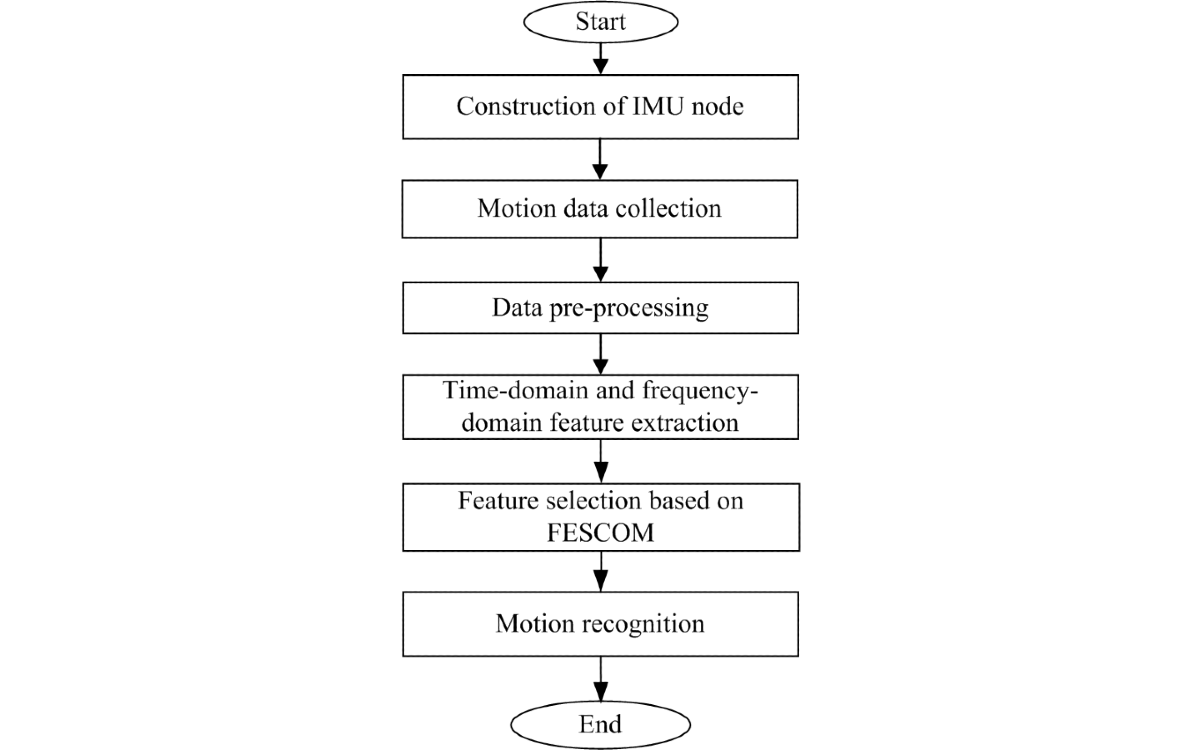
General workflow. FESCOM: hybrid feature selection method by combining filter and wrapper methods; IMU: inertial measurement unit.

The general workflow of this work is illustrated in Figure 1. An inertial measurement unit (IMU) node was customized for motion data collection. Motion data including acceleration and angular velocity from 5 types of upper-limb exercises were collected. Original data were preprocessed by applying a median filter to remove outliers. Time-domain and frequency-domain features were extracted from the preprocessed acceleration and angular velocity data on each axis. The feature selection method was built to select the most representative features for motion recognition. Then, motion recognition was implemented using the optimal feature set and corresponding classifier.

### Construction of the IMU

**Figure 2:**
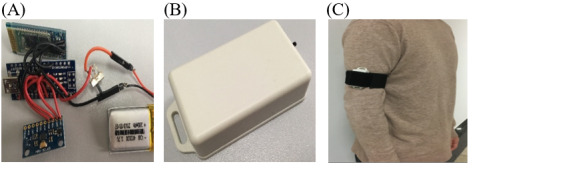
(A) inertial measurement unit (IMU) components, (B) box, and (C) belt.

The IMU module consists of 1 inertial sensor (MPU9250), 1 8-bit low power consumption micro-controller (ATmega328P on board nano V3.0), 1 Bluetooth wireless transmitter (HC-06), and 1 battery, shown in Figure 2. The inertial sensor MPU9250 is comprised of a 3-axis accelerometer, gyroscope, and magnetometer. The built-in digital motion processing engine in the MPU9250 can reduce the complex computation and load of the microcontroller. The measurement ranges of the accelerometer and gyroscope in the MPU9250 are ±16 *g* and ±2000 °/s, respectively, where *g* represents gravitational acceleration. These specifications meet the needs of upper-limb exercises. Sampled motion data can be transmitted to a PC station by Bluetooth in real time. The battery is 3.7 V and 200 mAh. No recharge module is used. It is convenient for wearable devices. The scale ranges of the accelerometer and the gyroscope can be adjusted using the programming interface of the inertial sensor and were set at ±2 *g* and ±250 °/s, respectively, in this study. The sampling frequency was set at 20 Hz, which is suitable for upper-limb exercises by patients with motion functionality impairment. The baud rate of Bluetooth was set at 19200 bps. Angular velocity was computed based on the gyroscope data. Magnetometer data were not used in this study. These components were connected and embedded into a 58 mm x 32 mm x 19 mm box. The IMU node was attached to the outside of the right upper limb of the participant with a stretchable 350 mm x 38 mm rubber belt, shown in Figure 2. The positive direction of the y-axis points to the wrist.

### Experimental Protocol

In this study, upper-limb exercises for post-stroke rehabilitation training were considered. From the clinical point of view, a subset of the training items can represent the 33 upper limb–related training items in the Fygl-Meyer Assessment (FMA) scale [37]. In this experiment, 5 representative upper-limb exercises based on the FMA scale were selected:

1. Forearm pronation and supination: Raise the right arm to the horizontal position in the sagittal plane. Then, carry out forearm pronation and supination.
2. Lumbar touch: The right arm hangs naturally. Move the right arm back to touch the back of the waist with the hand. Then, slowly move back to the initial position.
3. Shoulder touch: The right arm hangs naturally. Raise the right arm to the horizontal position in the sagittal plane. Then, carry out an elbow adduction motion and rotate the wrist to touch the opposite shoulder with the hand. Finally, put the arm down to the initial position.
4. Shoulder flexion: The right arm hangs naturally. Raise the right arm in the sagittal plane as high as possible. Then, hold for 3 seconds and move back to the initial position.
5. Shoulder extension: The right arm hangs naturally. Raise the right arm in the coronal plane as high as possible. Then, hold for 3 seconds and move back to the initial position.

**Figure 3:**
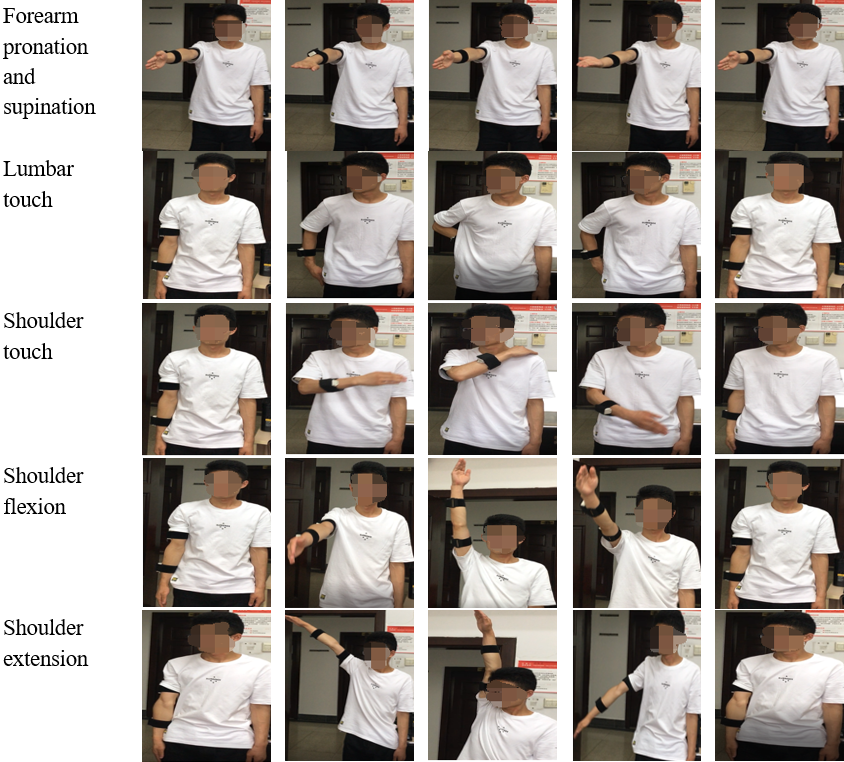
Sequence of each exercise performed by the participants.

Figure 3 includes 5 photos of each exercise taken during the execution process.

Motion data were collected from 21 healthy participants (15 men, 6 women; age, mean 33.2, SD 12.7 years; height, mean 172.5, SD 7.1 cm; weight, mean 62.8, SD 17.5 kg) instead of actual patients who are post-stroke. The study was approved by the institutional review board of the Eighth People’s Hospital of Chengdu. Written informed consent was obtained from all participants. In the sampling experiment, participants were first asked to rest for a while. Before the sampling began, they were invited to perform each exercise several times with the guidance of a guiding video until they performed the motions fluidly. Then, they were required to complete 3 valid repetitions of each exercise independently. Each repetition followed an interval of about 3 seconds. A valid repetition was a coherent movement, and each repetition was completed in 1-4 seconds.

### FESCOM Method

In this study, 10 types of time-domain and frequency-domain features were extracted from the motion data from the upper-limb exercises. The time-domain features included the mean, standard deviation, maximum, and minimum values of the signal as well as the kurtosis, skewness, and interquartile range of the signal, which may reflect the exercise frequency, regularity, and symmetry, respectively. The frequency-domain features included average power, average frequency, and median frequency of the signal. As each sample included acceleration and angular velocity data in 3 axes, the dimension of the original feature vector was 60.

**Figure 4:**
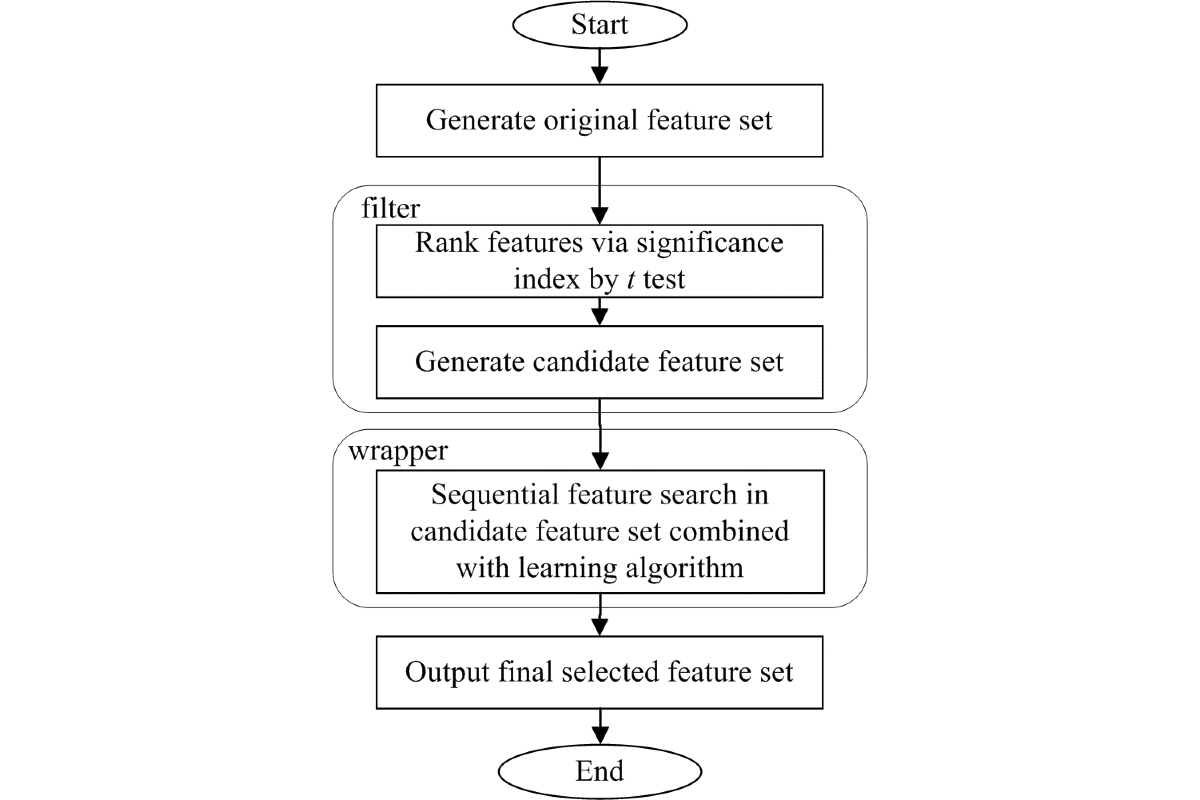
The hybrid feature selection method by combining filter and wrapper methods (FESCOM).

The original feature set contains not only the features that are relevant for classification but also some redundancy features, which decrease the computational efficiency and classification accuracy. We propose a hybrid feature selection method, called FESCOM, to remove redundant features so as to improve the computational efficiency and classification accuracy. Figure 4 shows the procedure of the FESCOM method. In the filter stage, the statistical *t* test method was adopted to compute the statistical significance value (*P* value) of each feature, reflecting the capability of motion recognition [38,39]. For samples *x* and *y*, a two-sample *t* test was considered for analysis, which is defined as:

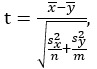



where 
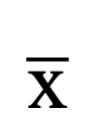
® and 
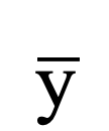
® are the sample means, *s_x_* and *s_y_* are the sample standard deviations, and *n* and *m* are the sample size. As there are 5 types of motion, the *t* test method was applied to each class pair. Let *P_k_*(*i*,*j*) represent the *P* value of feature *k* on class *i* and *j*, the average *P* value of feature *k* on all class pairs is computed as:

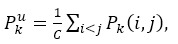



where *i*=1,…,*C*, *j*=*i*+1,…,*C*, and *C* is the number of motion classes. The standard deviation of the *P* value of feature *k* on all class pairs is:

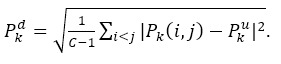



Then, the significance index of feature *k* is computed as:

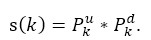



A smaller *s* value means stronger classification capacity. The features with *s* smaller than a threshold were selected for the candidate feature set, ranked in ascending order. The threshold value was a compromise between time efficiency and the classification accuracy of FESCOM.

**Figure 5:**
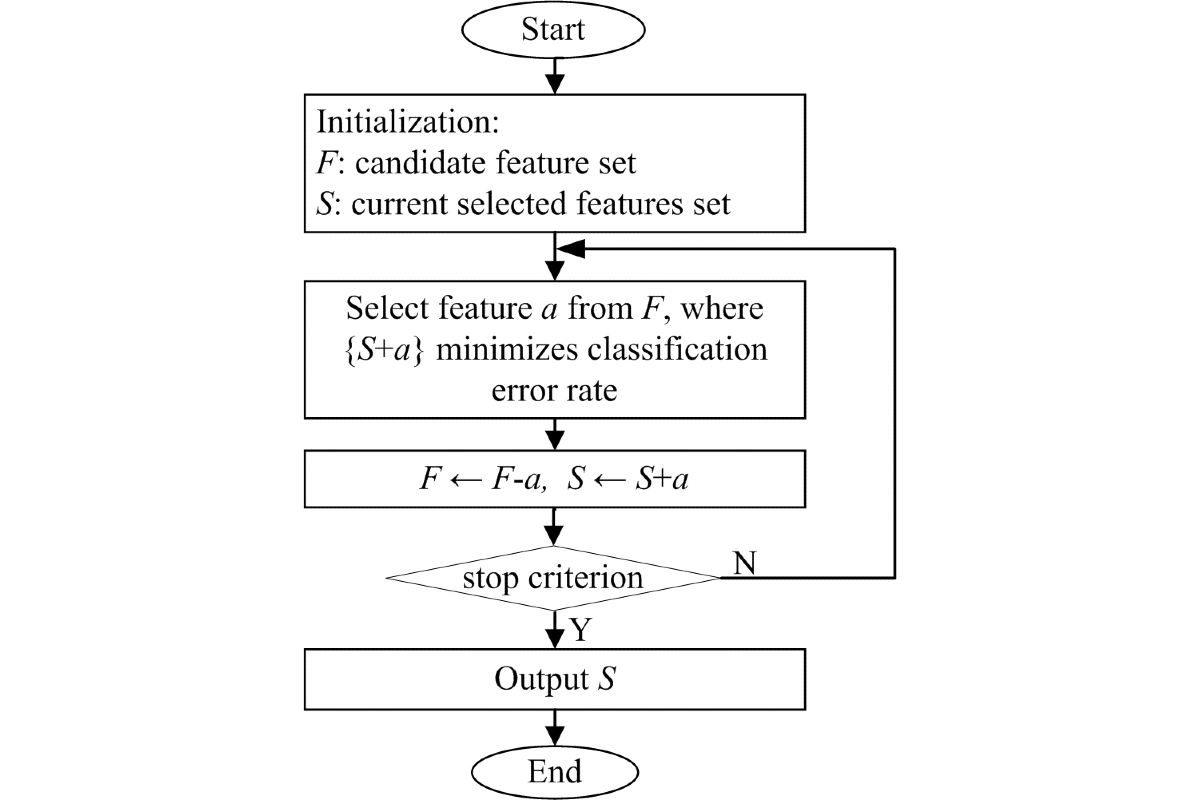
Sequential feature selection process.

In the wrapper stage, a sequential feature selection (SFS) method was used to refine the features from the candidate feature set obtained in the filter stage. SFS includes a search algorithm and an objective function, also called criterion [40]. In this study, the search algorithm was sequential forward selection, and the criterion was the classification error rate. Starting from an empty feature set, SFS selects a subset of features from the candidate feature set by sequentially selecting features using the abovementioned criterion until there is no improvement in classification performance, evaluated by each classification algorithm (ie, the wrapping component). The procedure of SFS is illustrated as Figure 5.

### Classification Algorithms

In the experiment, kNN, NB, and RF were adopted as classification algorithms in the wrapper stage of FESCOM.

Classifier kNN is a simple classiﬁcation algorithm based on the calculation of the distance (usually the Euclidean distance) between the new sample to be classiﬁed and the closest samples in the training set. The training samples are sorted in descending order according to their distance from the new object. Then, the new sample is assigned to the class that most of its k-nearest neighbors belong to [41].

NB is a probabilistic classifier based on the assumption that all features are independent of each other, given the category variable [42]. For discrete features, multinomial or Bernoulli distributions are popular. Despite apparently over-simplifier assumptions, NB classifier works quite well in many complex real-world applications, such as medical diagnosis, key phrase extraction, and text classification. The NB classifier is particularly useful for handling incomplete data and could yield good predictions even with a small data size.

RF is a type of ensemble learning method that is formed through the combination of multiple decision trees trained on the training dataset. When applied to the test dataset, the predictions of individual tree models within the RF are combined into an overall classiﬁcation decision through means of a majority vote or the application of weights. The RF model can avoid overﬁtting and provide robust classiﬁcation performances [43]. The number of decision trees in this experiment was set at 20.

## Results

### Overview

In this study, MATLAB 2016a was used to develop the proposed FESCOM method for motion recognition of upper-limb exercises. The original dataset was randomly partitioned into a training set and testing set. The training set was applied to train each classifier by using five-fold cross validation. For each iteration, one of the partitions was held back as the validation set, whereas the other partitions were used to train the classification model. The model was then validated by the validation set. This process was repeated 5 times, so that each subset was used as a validation set once. The results were averaged over all rounds. Finally, the performance of each classifier was evaluated on the testing set.

The performance of the proposed algorithm was evaluated using the metrics of classification error rate, computed as the ratio of number of instances classified incorrectly to the total number of instances.

### Experimental Data

**Figure 6:**
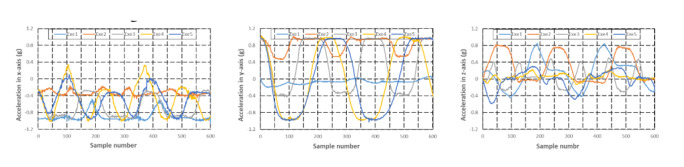
Acceleration in each axis of the 5 exercises.

**Figure 7:**
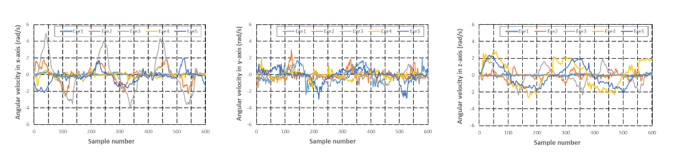
Angular velocity in each axis of the 5 exercises.

Acceleration and angular velocity in 3 axes of 5 exercises performed by 1 female participant are illustrated in Figure 6 and Figure 7, respectively. Exe1, Exe2, Exe3, Exe4, and Exe5 in Figures 6 and 7 represent the 5 types of motion defined in the experimental protocol section. The time-domain waveforms of the 5 exercises showed different characteristics. For example, the acceleration and angular velocity of forearm pronation and supination, lumbar touch, and shoulder touch showed totally different trends. Although the time-domain features of the acceleration in the y-axis of shoulder flexion were similar with those of shoulder extension, the acceleration in the x-axis of shoulder flexion exhibited a higher peak compared with that of shoulder extension. Moreover, angular velocity in both the x-axis and y-axis of shoulder flexion exhibited a smaller peak than that of shoulder extension. These differences between exercises can be used for motion recognition.

### Feature Significance Index

Table 1 shows the top-10 feature significance index value and rank order computed by the statistical *t* test method. An extended version of Table 1, including the significance index of all 60 features, is presented in Multimedia Appendix 1. The significance index value was computed on 2 types of signals (ie, acceleration and angular velocity), represented in parentheses following the feature name, with a suffix representing on which axis it is. The significance index value of the minimum angular_velocity_y ranks the highest, whereas the average acceleration_z ranks the lowest of all 60 features. A smaller significance index means stronger classification capacity.

**Table 1:** Top 10 feature significance index.

Feature name	Significance index	Rank order
minimum (angular_velocity_y)	0.00030	1
average power (angular_velocity_x)	0.00035	2
average power (acceleration_x)	0.00049	3
standard deviation (angular_velocity_z)	0.00058	4
skewness (acceleration_z)	0.00130	5
average power (acceleration_y)	0.00132	6
median frequency (acceleration_y)	0.00155	7
median frequency (angular_velocity_y)	0.00174	8
maximum (angular_velocity_x)	0.00240	9
standard deviation (angular_velocity_y)	0.00283	10

### Experimental Results

**Figure 8:**
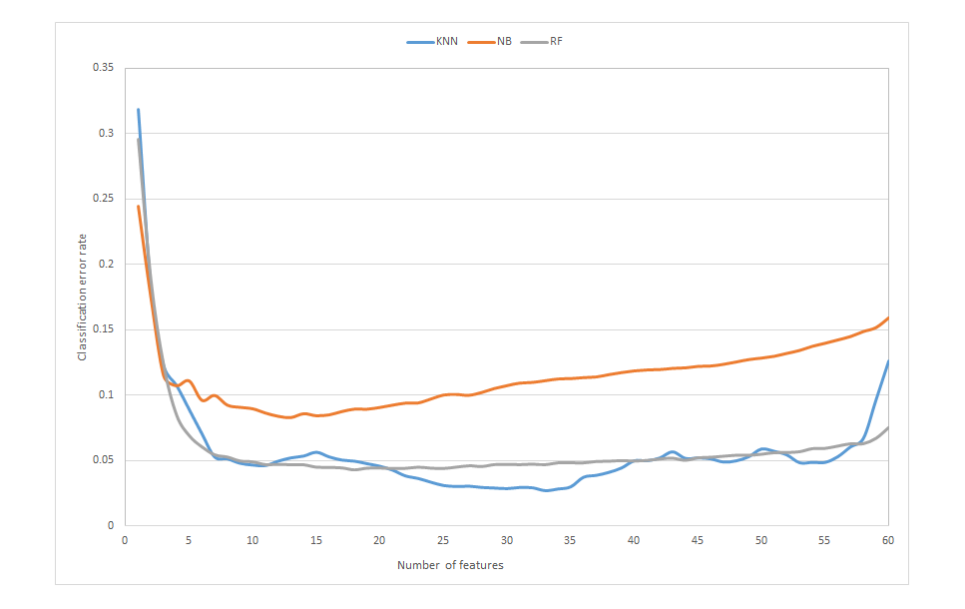
Classification error rate vs feature number. kNN: k-nearest neighbor; NB: Naïve Bayes; RF: random forest.

To analyze the impact of the feature number on the performance of motion recognition of upper-limb exercises, experiments were conducted on the training set including a different number of features. The results of the classification error rate are shown in Figure 8. The general trends for the 3 classifiers are similar. With an increase in feature number, the classification error rate decreases rapidly. With a further increase in feature number, the classification error rate shows an increasing trend. The trend for kNN is not as stable as that of NB and RF. For kNN, there are several local optima with an increase in feature number.

Table 2 shows the optimal number of features for the different classifiers. There is an obvious distinction between the optimal number of features for the different classifiers. Classifier kNN needs more features to achieve the minimum classification error rate than classifiers NB and RF.

**Table 2:** Optimal number of features.

Classifier	Optimal number of features
kNN^a^	33
NB^b^	13
RF^c^	18

^a^kNN: k-nearest neighbor.

^b^NB: Naïve Bayes.

^c^RF: random forest.

As wrapper methods usually achieve better classification performance than filter methods, and until now, there has been no hybrid feature selection method for motion recognition of rehabilitation exercises, we compared the motion recognition performance of the proposed FESCOM method with the traditional wrapper method. Experiments were conducted on the testing set by selecting the optimal feature set. For the traditional wrapper method, SFS was used to search for the optimal feature set from all 60 original features. For FESCOM, SFS was used to refine the features from the candidate feature set, composed of features with a significance index value smaller than 0.05 and ranked in ascending order. The criterion to set the threshold of the significance index was an assumption that the number of candidate features increased 30% (10 out of 33 features) from the highest optimal number of features in Table 2. The classification error rate is shown in Table 3. For both feature selection methods, the classification performance of kNN was better than that of NB and RF. The classification error rate of FESCOM using kNN and NB as the wrapping component was lower than the corresponding wrapper methods.

**Table 3:** Classification error rate.

Feature selection method	Classification error rate (%)		
	kNN^a^	NB^b^	RF^c^
			
Wrapper	2.2	13.4	6.7
FESCOM^d^	1.7	8.9	7.4

^a^kNN: k-nearest neighbor.

^b^NB: Naïve Bayes.

^c^RF: random forest.

^d^FESCOM: hybrid feature selection method by combining filter and wrapper methods.

The time consumed on feature selection in each iteration for both feature selection methods is listed in Table 4. As the candidate feature set of FESCOM is smaller than that of the wrapper method, the search time for FESCOM was much less than that of the wrapper method for all classifiers. For the same feature selection method, kNN needs much less search time than NB and RF.

**Table 4:** Search time for each iteration.

Feature selection method	Search time (seconds)		
	kNN^a^	NB^b^	RF^c^
			
Wrapper	23	159	876
FESCOM^d^	13	71	541

^a^kNN: k-nearest neighbor.

^b^NB: Naïve Bayes.

^c^RF: random forest.

^d^FESCOM: hybrid feature selection method by combining filter and wrapper methods.

## Discussion

### Principal Findings

This paper presents a hybrid feature selection method for motion recognition of upper-limb exercises. For motion recognition based on feature extraction and feature selection, the feature set used for the classification algorithm had a direct impact on the performance of classification. The experimental results in this study verified that recognition performance depends on the feature set. For all 3 classifiers in this study, the same trends existed: The classification error rate decreased to an optimum value when the number of features increased and increased with a further increase in feature number due to overfitting. The optimal number of features depended on the classifier. The optimal numbers of features for classifiers kNN, NB, and RF were 33, 13, and 18, respectively.

Each feature contributes differently to the motion classification task. Take the proposed FESCOM method as an example: The frequency-domain features contribute more than other features to recognition performance. When using the classifier kNN as the wrapping component, the top 3 significant features for motion recognition were average power of angular_velocity_x, average acceleration_x, and mean frequency of angular_velocity_x. When using the classifier NB as the wrapping component, the top 3 significant features for motion recognition were average power of acceleration_x, standard deviation of acceleration_y, and kurtosis of angular_velocity_x. When using the classifier RF as the wrapping component, the top 3 significant features for motion recognition were average power of angular_velocity_x, average power of acceleration_y, and mean frequency of angular_velocity_y.

Motion recognition performance also depends on the classifier. For both feature selection methods, the classification performance of kNN was the best, while NB was the worst classification performance. The proposed FESCOM method reduces the feature space and improves the time efficiency by filtering irrelevant features for motion classification.

### Comparison With Previous Works

The common methods for motion recognition combine wearable sensing techniques and machine learning algorithms. Acceleration, angular velocity, or sEMG signals collected by wearable sensors are used to represent the motion characteristics. Cai et al [8] exploited sEMG signals and the SVM classifier for motion recognition of upper-limb exercises; 5 healthy participants participated in the experiments. The average recognition accuracy of 5 motions was 93.34%. Motion recognition of upper-limb exercises in [15] was based on acceleration, angular velocity data, and BPNN algorithm; 13 volunteers participated in the experiments. Five upper-limb exercises involving simple swinging and stretching movements were recognized with an accuracy of 85%-95%, while exercises consisting of spiral rotations were recognized with an accuracy of 60%. The knowledge-driven activity recognition method in [9] focused on egocentric video and accelerometer/gyroscope data. Experiments were conducted on 3 public datasets, with a best recognition accuracy of 76.1%.

Using kNN, NB, and RF as the wrapping components, the recognition performance of FESCOM in this study achieved 98.3%, 91.1%, and 92.6%, respectively. Compared with previous studies on upper-limb motion recognition, the recognition performance of FESCOM is at the same level or even better than that in previous works. Time efficiency is one of the main concerns especially in real-time applications, such as motion recognition in autonomous rehabilitation systems. However, previous works seldom considered time efficiency. The FESCOM method in this study reduced the feature space and improved the time efficiency by filtering irrelevant features for motion classification. Compared with the search time of the traditional wrapper method, the search time of FESCOM using kNN, NB, and RF classifiers as the wrapping component reduced the search time by 43% (from 23 seconds to 13 seconds), 55% (from 159 seconds to 71 seconds), and 38% (from 876 seconds to 541 seconds), respectively. Hence, this study contributes by evaluating the number and types of features for different classification algorithms that achieve acceptable performance for motion recognition of upper-limb exercises.

### Limitations

The FESCOM method proposed in this study has some limitations. It was only evaluated based on data from 21 healthy participants, and only 5 types of upper-limb exercises were considered in the experiments. However, the behavior of patients with a central nervous system lesion, such as that caused by stroke, may be very different from that of healthy participants. The experimental results may be different in such cases. The number of samples for training and testing is not high enough for machine learning algorithms, which may also affect the reliability. The customized IMU module in this work is just a prototype. The components in the sensor node are connected with cables. This may lead to unreliable connections, especially when used in movement conditions. Another drawback is that the validation of the system did not use real-time exercise examples.

In our future work, to further confirm the feasibility of FESCOM, we plan to extend our experiment considering the following aspects. First, we will evaluate and compare the performance of different methods in the filter and wrapper stage of FESCOM. Second, we will evaluate the performance of FESCOM considering more classifiers as the wrapping component in the wrapper stage, such as SVM and latent Dirichlet allocation. Third, we will evaluate the performance of FESCOM on more datasets, such as public datasets including more motion types and datasets including not only healthy participants but also real patients with different functional impairments in the recovery stage in a clinical situation. Fourth, we plan to improve the IMU node as an embedded system on a circuit board for real-time data collection and validate the whole system by real-time prediction of upper-limb exercises.

### Conclusions

In this study, a hybrid feature selection method, FESCOM, was proposed for motion recognition of upper-limb exercises and evaluated using 5 types of upper-limb exercises performed by 21 healthy participants. The experimental results demonstrate that FESCOM is feasible for motion recognition of upper-limb exercises performed by healthy participants. FESCOM improves the recognition accuracy when using kNN and NB as the wrapping component and improves the time efficiency in the wrapper stage. The results also demonstrate that, for different classifiers, different feature sets are selected to achieve optimal performance. This work can be extended to provide motion recognition of more motion types and participants including healthy people and actual patients with minor motor damage.

## Appendix

Multimedia Appendix 1 Extended version of Table 1 with significance index of all 60 features.

## Abbreviations

BPNN: back-propagation neural network
FESCOM: hybrid feature selection method by combining filter and wrapper methods
FMA: Fygl-Meyer Assessment
IMU: inertial measurement unit
kNN: k-nearest neighbor
MGRA: motion gesture recognition system via accelerometer
NB: Naïve Bayes
RF: random forest
sEMG: surface electromyogram
SFS: sequential feature selection
SVM: support vector machine
