# Supplementary material for: Upper-Limb Motion Recognition Based on Hybrid Feature Selection: Algorithm Development and Validation
Source: JMIR Mhealth Uhealth. 2021 Sep 2;9(9):e24402. doi: 10.2196/24402 (PMC8446846; doi:10.2196/24402)
Supplement: Multimedia Appendix 1 [file mhealth_v9i9e24402_app1.docx]

Extended version of Table 1 with significance index of all 60 features.

| Feature name | Significance index | Rank order |
| --- | --- | --- |
| minimum (angular_velocity_y) | 0.00030 | 1 |
| average power (angular_velocity_x) | 0.00035 | 2 |
| average power (acceleration_x) | 0.00049 | 3 |
| standard deviation (angular_velocity_z) | 0.00058 | 4 |
| skewness (acceleration_z) | 0.00130 | 5 |
| average power (acceleration_y) | 0.00132 | 6 |
| median frequency (acceleration_y) | 0.00155 | 7 |
| median frequency (angular_velocity_y) | 0.00174 | 8 |
| maximum (angular_velocity_x) | 0.00240 | 9 |
| standard deviation (angular_velocity_y) | 0.00283 | 10 |
| maximum (angular_velocity_z) | 0.00439 | 11 |
| standard deviation (acceleration_x) | 0.00439 | 12 |
| standard deviation (acceleration_z) | 0.00784 | 13 |
| minimum (acceleration_z) | 0.00859 | 14 |
| kurtosis (angular_velocity_y) | 0.00887 | 15 |
| interquartile range (acceleration_z) | 0.01140 | 16 |
| mean frequency (angular_velocity_x) | 0.01191 | 17 |
| interquartile range (acceleration_x) | 0.01231 | 18 |
| minimum (angular_velocity_z) | 0.01370 | 19 |
| kurtosis (angular_velocity_z) | 0.01572 | 20 |
| mean frequency (acceleration_x) | 0.01658 | 21 |
| minimum (angular_velocity_x) | 0.01763 | 22 |
| interquartile range (acceleration_y) | 0.01837 | 23 |
| median frequency (acceleration_x) | 0.02073 | 24 |
| standard deviation (acceleration_y) | 0.02219 | 25 |
| average power (angular_velocity_z) | 0.02268 | 26 |
| interquartile range (angular_velocity_x) | 0.02311 | 27 |
| average­ (acceleration_x) | 0.02335 | 28 |
| average (angular_velocity_z) | 0.02354 | 29 |
| average (acceleration_y) | 0.02436 | 30 |
| mean frequency (acceleration_y) | 0.02461 | 31 |
| skewness (angular_velocity_y) | 0.02670 | 32 |
| standard deviation (angular_velocity_x) | 0.02681 | 33 |
| kurtosis (acceleration_y) | 0.02697 | 34 |
| median frequency (angular_velocity_x) | 0.03132 | 35 |
| maximum (acceleration_z) | 0.03133 | 36 |
| skewness (angular_velocity_x) | 0.03264 | 37 |
| maximum (acceleration_y) | 0.03412 | 38 |
| skewness (acceleration_y) | 0.03632 | 39 |
| average power (acceleration_z) | 0.03828 | 40 |
| mean frequency (angular_velocity_y) | 0.04524 | 41 |
| maximum (acceleration_x) | 0.04552 | 42 |
| kurtosis (angular_velocity_x) | 0.04881 | 43 |
| median frequency (angular_velocity_z) | 0.05073 | 44 |
| average power (angular_velocity_y) | 0.05274 | 45 |
| median frequency (acceleration_z) | 0.05644 | 46 |
| minimum (acceleration_y) | 0.05742 | 47 |
| average (angular_velocity_x) | 0.06068 | 48 |
| mean frequency (angular_velocity_z) | 0.06091 | 49 |
| interquartile range (angular_velocity_y) | 0.06190 | 50 |
| interquartile range (angular_velocity_z) | 0.06954 | 51 |
| mean frequency (acceleration_z) | 0.08316 | 52 |
| verage (angular_velocity_y) | 0.08888 | 53 |
| maximum (angular_velocity_y) | 0.09324 | 54 |
| skewness (angular_velocity_z) | 0.09360 | 55 |
| kurtosis (acceleration_z) | 0.09480 | 56 |
| kurtosis (acceleration_x) | 0.09874 | 57 |
| skewness (acceleration_x) | 0.10542 | 58 |
| minimum (acceleration_x) | 0.12177 | 59 |
| average (acceleration_z) | 0.13761 | 60 |
